# Prevalence and clinical relevance of sarcopenia in Chinese patients with porto-sinusoidal vascular disorder

**DOI:** 10.3389/fmed.2025.1756842

**Published:** 2026-01-12

**Authors:** Jiaxin Chen, Xin Quan, Shuaijie Qian, Yang Tai, Zhidong Wang, Huan Tong, Jinhang Gao, Bo Wei, Hao Wu

**Affiliations:** Department of Gastroenterology and Hepatology, West China Hospital, Sichuan University, Chengdu, China

**Keywords:** porto-sinusoidal vascular disorder (PSVD), sarcopenia, portal hypertension, esophagogastric variceal bleeding (EGVB), rebleeding

## Abstract

**Aim:**

Porto-sinusoidal vascular disorder (PSVD) is a rare vascular liver disease that has similar manifestations to liver cirrhosis. However, limited information is available on the prevalence and clinical impacts of sarcopenia in patients with PSVD in China.

**Methods:**

We enrolled 92 patients with PSVD from West China Hospital between January 2019 to June 2024 to analyze and characterize the burden of sarcopenia. The psoas muscle mass index (PMI), measured at the third lumbar vertebra (L3-PMI, cm^2^/m^2^), was used for quantitative analysis of skeletal muscle mass. Age- and sex- specific cut-off values, optimized for the Chinese population, were endorsed for the diagnosis of sarcopenia.

**Results:**

Sarcopenia was observed in 17 out of the 92 PSVD patients (18.5%). The sarcopenia group had a higher proportion of patients with a history of esophagogastric variceal bleeding (EGVB) compared to the non-sarcopenia group (88.24% vs. 60.0%, *p* = 0.046). Transjugular intrahepatic portosystemic shunt (TIPS) creation significantly increased L3-PMI [4.84 (3.70–5.44) vs. 5.10 (4.40–6.10), *p* = 0.0153]. During the 1-year follow-up after endoscopic treatment, the cumulative incidence of rebleeding was significantly higher in the sarcopenia group than in the non-sarcopenia group [HR 4.39, 95% CI 1.01–19.10, log-rank *p* = 0.0485]. Multivariate Cox regression analysis showed that sarcopenia was an independent predictor of 1-year variceal rebleeding.

**Conclusion:**

Sarcopenia is common in patients with PSVD, particularly among those with a history of EGVB. TIPS placement may potentially enhance the muscle mass of PSVD patients. Importantly, sarcopenia appears to negatively impact clinical outcomes in PSVD patients undergoing endoscopic therapy for variceal bleeding.

## Introduction

Sarcopenia, a key nutritional parameter, is defined as the generalized loss of skeletal muscle mass, strength and function attributable to aging (primary sarcopenia) or to acute or chronic illness (secondary sarcopenia), with cirrhosis being a common precipitant (1, 2). Multiple factors, including reduced nutrient intake, altered protein and energy metabolism, maldigestion and malabsorption resulting from portal hypertension-induced gastrointestinal congestion, and additional factors such as infections and systemic inflammation contribute to malnutrition in cirrhosis (3).

Porto-sinusoidal vascular disorder (PSVD) is a rare vascular liver disorder. Based on the evidence in cirrhotic patients, where sarcopenia is an established predictor independently of the degree of liver dysfunction (4, 5), we hypothesize that sarcopenia may similarly influence clinical outcomes in PSVD, which is characterized by well-preserved hepatic function but may develop severe portal hypertension (6). Specifically, esophagogastric variceal bleeding (EGVB) is a major complication of portal hypertension at the time of PSVD diagnosis (6, 7). According to current guidelines, the combination of endoscopic treatment and non-selective beta-blockers (NSBBs) is the first-line therapy for EGVB, with transjugular intrahepatic portosystemic shunt (TIPS) reserved as a rescue option, regardless of whether it is for primary or secondary prophylaxis (8, 9). Notably, previous studies have demonstrated that sarcopenia is an independent risk factor for worsened clinical outcomes in cirrhotic patients after endoscopic therapy or TIPS placement, including rebleeding, overt hepatic encephalopathy (OHE), and even death (10–12). However, to our knowledge, little is known regarding the prevalence of sarcopenia in patients with PSVD and its impact on clinical outcomes.

Considering the recognized importance of assessing sarcopenia in the management of cirrhotic patients, as emphasized in the Baveno VII consensus, the impact of sarcopenia on patients with PSVD and portal hypertension needs to be determined (8). In this study, we retrospectively collected clinical and radiological data from PSVD patients followed at West China Hospital, Sichuan University. We aimed to determine the prevalence of sarcopenia in this cohort and evaluate its clinical implications.

## Methods

### Patients and baseline characteristics

This retrospective study was approved by the Ethics Committee of West China Hospital, Sichuan University (protocol number: 2022–1680). A total of 92 PSVD patients who had available abdominal computed tomography (CT) images and were followed in our department from January 2019 to June 2024 were included. PSVD was diagnosed according to the criteria set forth in the Baveno VII consensus: absence of cirrhosis and of any other known causes of portal hypertension on high-quality liver biopsies, plus 1 of the 3 conditions: (1) at least 1 sign specific to portal hypertension (gastric, esophageal, or ectopic variceal; portal hypertensive bleeding; portosystemic collaterals at imaging); or (2) at least 1 histological lesion characteristic of PSVD (obliterative portal venopathy; nodular regenerative hyperplasia; incomplete septal fibrosis or cirrhosis); or (3) at least 1 non-specific feature of portal hypertension (ascites; platelet count <150,000/mm^3^; spleen size ≥13 cm in the largest axis) together with a histological lesion not specific for PSVD (portal tract abnormalities; architectural disturbance; non-zonal sinusoidal dilatation; mild perisinusoidal fibrosis) (8). Patients with portal vein thrombosis (PVT) were enrolled only if there was clear evidence that portal hypertension had developed prior to PVT, or if specific histological features of PSVD were identified. All liver biopsy specimens were evaluated by expert pathologists. The classification of ascites based on the guidance from the American Association for the Study of Liver Diseases (13). Baseline clinical and laboratory characteristics were retrospectively extracted from data obtained at the time of each patient’s first hospitalization.

### Sarcopenia diagnosis

The cross-sectional psoas muscle area (PMA, cm^2^) was measured at the level of the third lumbar vertebra (L3) on a single axial image, as this region correlates closely with skeletal muscle area (SMA) and can be more simply evaluated with established reference values among a healthy Chinese population (14). The PMA was manually delineated using syngo.via imaging software (Siemens Healthineers, Erlangen, Germany). Three trained observers (JC, XQ, and SQ) identified each patient’s transverse CT image blinded to the patients’ clinical information, and the mean of the three measurements was used for analysis. The psoas muscle index (PMI) was calculated by normalizing the PMA to the square of the patient’s height (cm^2^/m^2^). Sarcopenia was diagnosed using age- and sex-specific L3-PMI thresholds validated in healthy Chinese population. Using the 5th-percentile cut-offs maximized sensitivity: for males, the thresholds were 5.41 cm^2^/m^2^ for 20–29 years, 4.71 cm^2^/m^2^ for 30–39 years, 4.65 cm^2^/m^2^ for 40–49 years, 4.10 cm^2^/m^2^ for 50–59 years and 3.68 cm^2^/m^2^ for over 60 years; the corresponding values for females were 3.32, 3.40, 3.18, 2.91, and 2.62 cm^2^/m^2^, respectively (14).

### Follow-up and outcomes

All patients were followed up until June 2025, liver transplantation or death. Those who presented with EGVB received standard therapy or underwent TIPS placement. Standard therapy consisted of endoscopic treatment (including endoscopic variceal ligation or cyanoacrylate injection) plus propranolol, starting at 10 mg/d and titrated to the maximum tolerated dose to achieve either a 25% reduction in resting heart rate or a target rate of 50–60 beats per minute. Regular endoscopic surveillance was carried out, and, if indicated, sequential endoscopic treatment was undertaken. Experienced interventional radiologists within our team carried out the TIPS procedure using an 8-mm covered stent (Fluency; Bard), with the aim of achieving a portosystemic pressure gradient of either < 12 mmHg or a decrement > 20% from baseline. Patency of stent was routinely assessed by Doppler ultrasound and abdominal CT every 6 months. Patients who had undergone TIPS intervention were assessed for changes in L3-PMI at 6 months after TIPS.

### Statistical analysis

Analysis was performed using SPSS software (version 24.0) and GraphPad Prism (version 8.0). Continuous variables were reported as medians [inter quartile range (IQR)] and were compared using Student’s *t*-tests or non-parametric tests, as appropriate. Categorical variables were shown as frequencies (percentages) and compared using Chi-square or Fisher’s exact test. Kaplan-Meier analysis was carried out to compare the cumulative incidence of rebleeding or OHE between groups stratified by treatment strategy and sarcopenia status. To compare the cumulative incidence curves of the two groups, a log-rank test was performed. To identify risk factors of variceal rebleeding after standard treatment, multivariable Cox regression analysis was performed. A paired-sample *t*-test was used to assess the difference in L3-PMI before and after treatment of TIPS. Statistical significance was defined as a two-sided *p* value of <0.05.

## Results

### PSVD population

**Figure 1:**
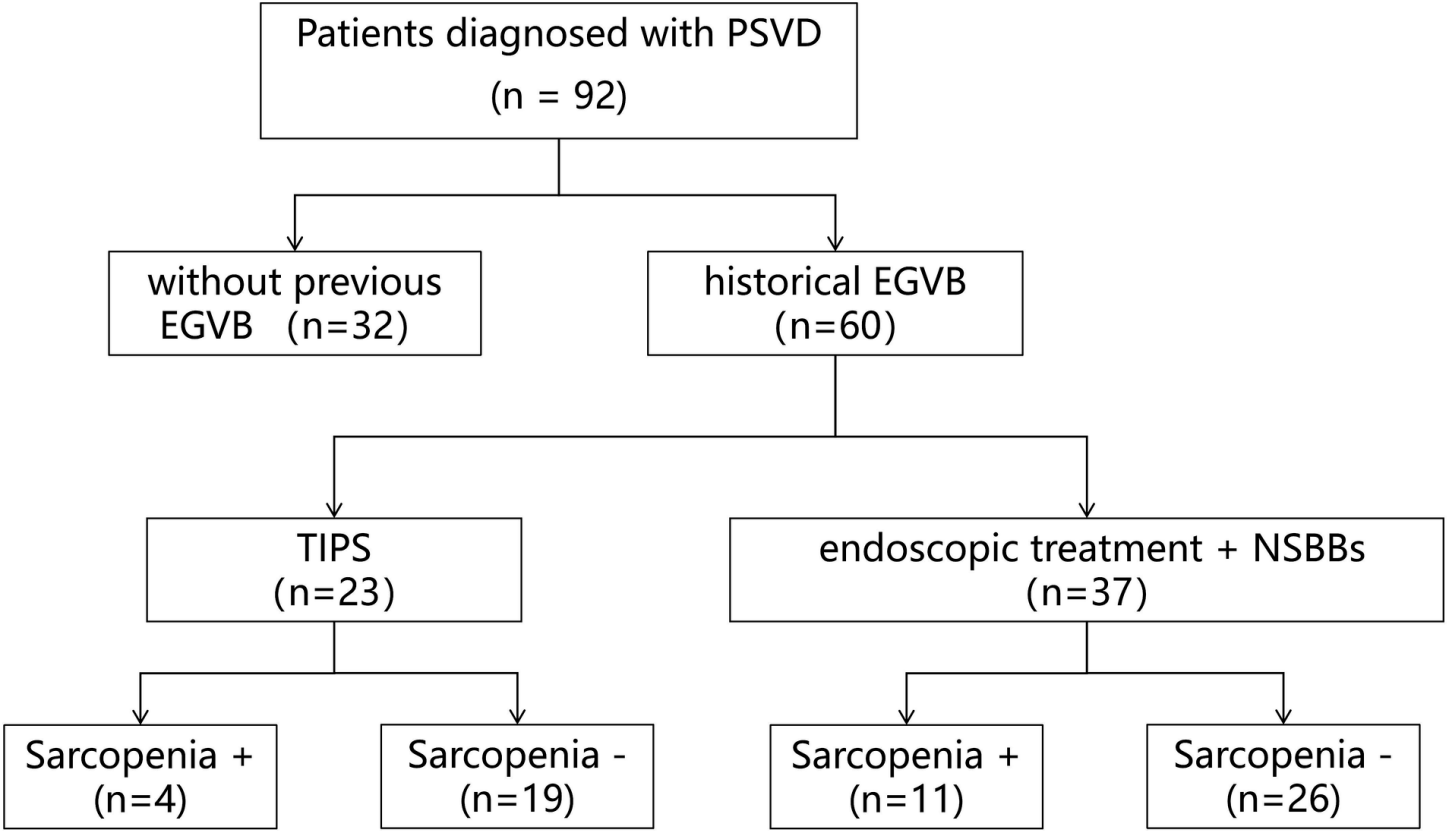
Research flowchart of this study. EGVB, esophagogastric variceal bleeding; NSBBs, non-selective beta-blockers; PSVD, porto-sinusoidal vascular disorder; TIPS, transjugular intrahepatic portosystemic shunt.

Between January 2019 to June 2024, 92 PSVD patients followed at the Department of Gastroenterology, West China Hospital of Sichuan University with evaluable abdominal CT images were enrolled. Figure 1 illustrates the study flowchart. According to the diagnosed criteria set forth in the Baveno VII consensus, 81 patients had specific signs of portal hypertension, whereas 11 patients had non-specific features. Pathologically, 43 patients had histological lesions characteristic of PSVD (predominantly obliterative portal venopathy), while the others had non-specific histological lesions (mainly portal tract abnormalities and non-zonal sinusoidal dilatation). A total of 12 patients with significant portal hypertension developed PVT during follow-up, although none had PVT at the time of initial diagnosis. At the time of PSVD diagnosis, 26 patients (28.26%) were asymptomatic: 20 were detected during routine physical examination showing splenomegaly with or without thrombocytopenia, and 6 were identified incidentally through abnormal results of liver-function tests. Among the 66 symptomatic patients (71.74%), 46 presented with isolated variceal bleeding, 6 with isolated ascites, and 14 with concomitant variceal bleeding and ascites. The mean age of all patients was 56 (41–65) years, and the proportion of males and females was approximately equal (44 males, 48 females). As shown in Table 1, no associated conditions were identified in 51 patients (55.43%). Conversely, underlying associated conditions were found in the remaining 41 patients (44.57%), with exposure to antineoplastic drugs (*n* = 12, 13.04%) being the most frequent, followed by PAI-1 4G/5G (*n* = 5, 5.43%), idiopathic thrombocytopenic purpura (*n* = 4, 4.35%) and Protein C/Protein S deficiency (*n* = 4, 4.3%).

**Table 1:** Underlying associated conditions in 92 PSVD patients.

Associated disorders	Overall (*n* = 92)
No associated condition identified	51 (55.43)
Common variable immune deficiency	3 (3.26)
Rheumatoid arthritis	2 (2.17)
Psoriasis	1 (1.11)
Grave’s disease	1 (1.11)
Type 1 diabetes mellitus	1 (1.11)
Sjögren’s sydrome	1 (1.11)
Elevated serum IgG4	1 (1.11)
Myeloproliferative neoplasm	1 (1.11)
Idiopathic thrombocytopenic purpura	4 (4.35)
Myelofibrosis	1 (1.11)
Aplastic anemia	1 (1.11)
Protein C/Protein S deficiency	4 (4.35)
PAI-1 4G/5G	5 (5.43)
Gilbert syndrome	2 (2.17)
Hyper IgE sydrome	1 (1.11)
Exposure to antineoplastic drugs	12 (13.04)

Data are presented as n (%). IgE, immunoglobulin E; IgG, immunoglobulin G; PAI-1, plasminogen activator inhibitor-1.

### Sarcopenia prevalence and baseline characteristics

Baseline characteristics are shown in Table 2. Among the 92 patients with PSVD included in this study, the prevalence of sarcopenia was 18.5% (17 of 92) based on L3-PMI cut-offs. The mean age of patients with sarcopenia (Sar+) was significantly lower than that of those without sarcopenia (Sar-) (*p* = 0.032). In the Sar + group, both L3-PMA [9.20 (6.05–10.40) vs. 13.40 (10.50–17.60), *p* < 0.001] and L3-PMI [3.26 (2.24–3.83) vs. 5.13 (4.15–6.10), *p* < 0.001] were significantly lower compared with baseline in the Sar- group. Likewise, both hemoglobin [73 (63–94) vs. 93 (74–113), *p* = 0.019] and white blood cell counts [2.19 (1.61–3.16) vs. 3.04 (2.13–4.55), *p* = 0.04] were significantly lower in the Sar + group.

**Table 2:** Baseline characteristics in PSVD patients with and without sarcopenia.

Variable	Overall (*n* = 92)	Sarcopenia + (*n* = 17)	Sarcopenia − (*n* = 75)	*p*-value
Age (years)	56 (41–65)	46 (23–75)	57 (44–65)	0.032
Gender (male)	44 (47.82)	9 (52.94)	35 (46.67)	0.789
Associated disorder	41 (44.57)	6 (35.29)	35 (46.67)	0.432
Height (m)	1.62 (1.56–1.67)	1.65 (1.61–1.68)	1.60 (1.55–1.66)	0.181
L3-PMA (cm^2^)	11.95 (9.20–16.00)	9.20 (6.05–10.40)	13.40 (10.50–17.60)	<0.001
L3-PMI (cm^2^/m^2^)	4.80 (3.51–5.73)	3.26 (2.24–3.83)	5.13 (4.15–6.10)	<0.001
Hemoglobin (g/L)	89 (71–108)	73 (63–94)	93 (74–113)	0.019
WBC (×10^9^/L)	2.91 (1.92–4.26)	2.19 (1.61–3.16)	3.04 (2.13–4.55)	0.04
Platelet (×10^9^/L)	65 (41–97)	53 (47–84)	65 (35–100)	0.637
Total bilirubin (μmol/L)	15.10 (11.03–22.53)	13.70 (9.75–24.55)	15.02 (11.20–22.70)	0.89
Albumin (g/L)	38.55 (33.95–42.58)	39.20 (33.90–43.05)	38.50 (33.90–42.60)	0.669
Creatinine (μmol/L)	68 (57–85)	63 (53–85)	70 (57–85)	0.839
INR	1.16 (1.06–1.28)	1.25 (1.07–1.28)	1.13 (1.06–1.29)	0.744
Child-Pugh score	6 (5–7)	6 (5–8)	6 (5–7)	0.312
Child-Pugh A	55 (59.78)	10 (58.82)	45 (60.00)	0.929
Child-Pugh B	37 (40.22)	7 (41.18)	30 (40.00)	
MELD score	8.10 (6.41–10.38)	9.03 (6.84–11.37)	7.52 (6.35–10.31)	0.879
Ascites (moderate to severe)	20 (21.73)	7 (41.18)	13 (17.33)	0.048
Previous variceal bleeding	60 (65.22)	15 (88.24)	45 (60.00)	0.046

Data are presented as n (%) or median (IQR). INR, international normalized ratio; MELD, Model for End-Stage Liver Disease; L3-PMA, psoas muscle mass area taken at the third lumbar vertebra; L3-PMI, psoas muscle mass index taken at the third lumbar vertebra; WBC, white blood cell.

**Figure 2:**
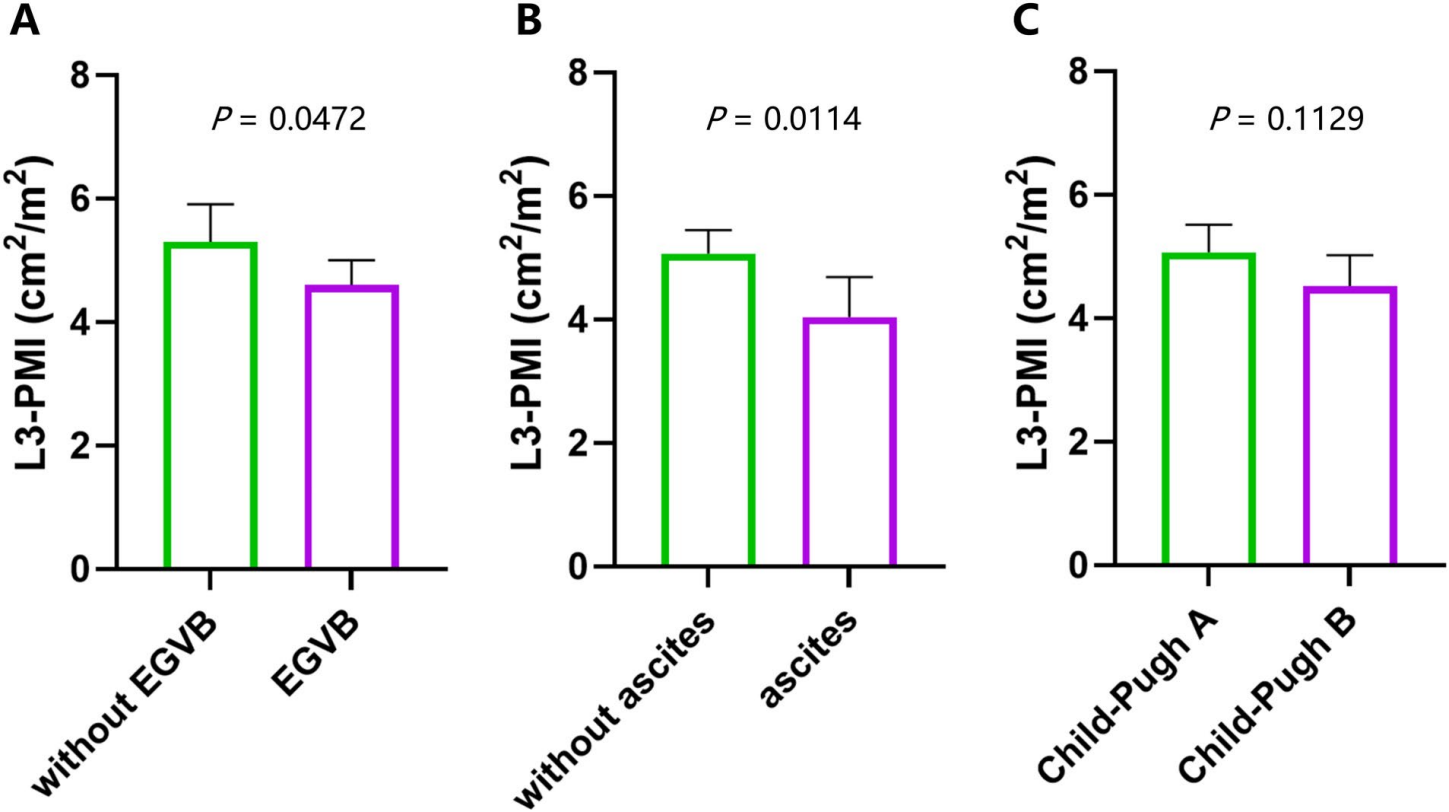
Comparison of L3-PMI among 92 patients with and without previous EGVB **(A)**, moderate to severe ascites **(B)**, and different Child-Pugh classes **(C)**.

No significant differences were observed in Child-Pugh scores and Model for End-stage Liver Disease (MELD) scores between the Sar + group and the Sar- group. In contrast, the prevalence of moderate-to-severe ascites was significantly higher in the Sar + group compared to the Sar- group (41.18% vs. 17.33%, *p* = 0.048). Given the comparable prevalence of associated disorder, the excess burden of ascites observed in the Sar + group seems more strongly associated with portal hypertension and/or sarcopenia than with oncologic or rheumatologic etiologies. Notably, 15 of 17 patients in the Sar + group and 45 of 75 patients in the Sar- group had a history of variceal bleeding (88.24% vs. 60.00%, *p* = 0.046). Significantly lower L3-PMI was associated with the presence of decompensation events (EGVB, ascites) but not with a worse Child-Pugh stage (Figure 2). These results indicated that muscle loss is more strongly associated with portal hypertensive complications than with liver dysfunction in PSVD patients.

### Clinical outcomes after endoscopic or TIPS treatment

**Figure 3:**
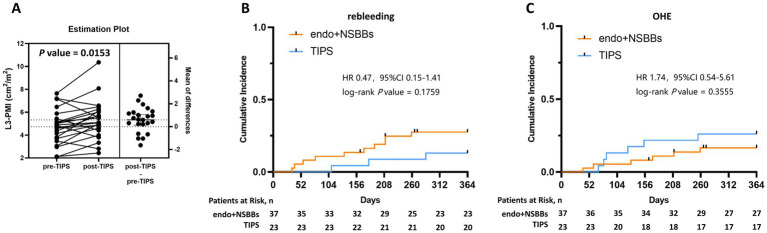
Changes of L3-PMI among 23 PSVD patients before and 6 months after TIPS **(A)**. The cumulative incidence of the variceal rebleeding **(B)** and the first episode of OHE **(C)** within 1 year after enrollment per treatment group. OHE, overt hepatic encephalopathy.

At the diagnosis of PSVD, 60 patients have a history of EGVB. A total of 23 patients in our cohort underwent TIPS for secondary prophylaxis of EGVB. In contrast, the remaining 37 patients with EGVB received endoscopic treatment. As shown in Figure 3A, TIPS placement in 23 EGVB patients was associated with a significant rise in L3-PMI [4.84 (3.70–5.44) vs. 5.10 (4.40–6.10), *p* = 0.0153] 6 months later, suggesting that TIPS exerts a beneficial effect on sarcopenia in the PSVD population. In addition, we compare the cumulative incidence of 1-year rebleeding and OHE between patients who received standard endoscopic therapy and those who underwent TIPS placement. As shown in Figures 3B,C, the endoscopy group showed a non-significant trend toward a higher incidence of 1-year rebleeding, whereas the TIPS group had a non-significant trend toward a higher incidence of in 1-year OHE.

### Impact of sarcopenia on clinical outcomes after endoscopic or TIPS therapy

**Figure 4:**
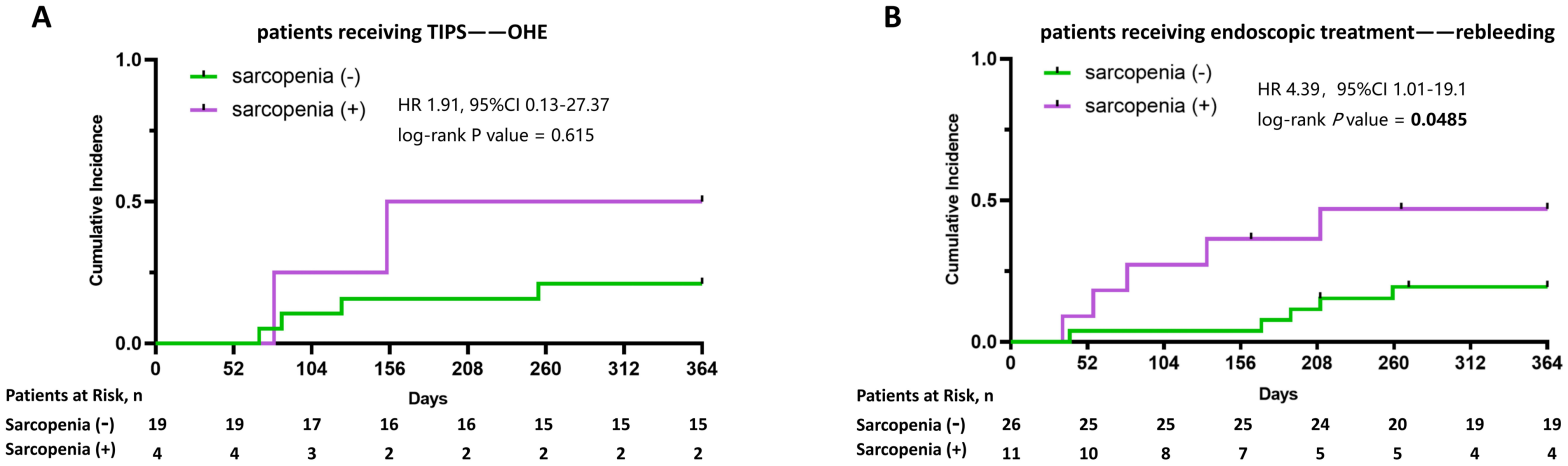
The cumulative incidence of the first episode of OHE within 1 year among patients receiving TIPS with and without sarcopenia **(A)**. The cumulative incidence of the variceal rebleeding within 1 year among patients receiving endoscopic treatment with and without sarcopenia **(B)**.

To investigate the clinical relevance of sarcopenia in PSVD patients after endoscopic or TIPS treatment, we performed subgroup analysis for each treatment group. In 23 PSVD patients receiving TIPS treatment, 4 patients had sarcopenia. No significant difference was observed in the cumulative incidence of OHE between the Sar- and Sar + groups (Figure 4A). For 37 PSVD patients receiving endoscopic therapy, 11 patients had sarcopenia. During a 1-year follow-up, variceal rebleeding occurred in 10 patients (27.03%) in the endoscopy group. The Sar + group had a significantly higher cumulative incidence of 1-year rebleeding compared to the Sar- group (HR 4.39, 95% CI 1.01–19.10, log-rank *p* = 0.0485), as shown in Figure 4B. Multivariable Cox regression analysis was performed to identify risk factors for variceal rebleeding, as shown in Table 3. Sarcopenia was identified as an independent predictor of 1-year variceal rebleeding, with fully adjusted odds ratios of 6.20 (95% CI 1.23–31.15, *p* = 0.027) in model 1 and 6.32 (95% CI 1.50–26.66, *p* = 0.012) in model 2, after adjusting for age and liver function.

**Table 3:** Risk factors of variceal rebleeding in multivariable Cox regression analyses.

Model 1			Model 2		
Variable	OR (95% CI)	*p*-value	Variable	OR (95% CI)	*p*-value
Age	1.04 (0.99–1.09)	0.057	Age	1.04 (0.99–1.08)	0.053
Albumin	1.00 (0.85–1.18)	0.996	Creatinine	1.00 (0.99–1.00)	0.345
Ascites	1.89 (0.19–18.64)	0.587	Child-Pugh score	1.33 (0.75–2.38)	0.334
MELD score	1.06 (0.93–1.21)	0.389	Sarcopenia	6.32 (1.50–26.66)	0.012
Sarcopenia	6.20 (1.23–31.15)	0.027			

Analyses is based on 37 patients with previous esophagogastric variceal bleeding and receiving endoscopic treatment.

Other complications are shown in Table 4. A total of 3 patients died within 1 year: 2 patients died from progression of the primary underlying disease and sepsis, and 1 died from fatal variceal rebleeding.

**Table 4:** Summary of outcomes during follow-up, stratified by treatment group and presence of sarcopenia.

Outcome	Endoscopic + NSBBs		TIPS	
	Sarcopenia**+** (*n* = 11)	Sarcopenia**−** (*n* = 26)	Sarcopenia**+** (*n* = 4)	Sarcopenia**−** (*n* = 19)
Variceal rebleeding	5 (45.5)	5 (19.2)	1 (25)	2 (10.5)
OHE	2 (18.2)	4 (15.4)	2 (50)	4 (21.1)
Shunt occlusion	–	–	0 (0)	2 (10.5)
Infections/Sepsis	1 (9.1)	2 (7.7)	1 (25)	2 (10.5)
ACLF	0 (0)	0 (0)	0 (0)	0 (0)
Liver transplantation	0 (0)	0 (0)	0 (0)	0 (0)
Death	1 (9.1)	1 (3.8)	1 (25)	0 (0)

Data are presented as n (%). OHE, overt hepatic encephalopathy; ACLF, acute-on-chronic liver failure.

## Discussion

Malnutrition is strongly associated with adverse clinical outcomes. A nationwide cross-sectional study reported that approximately 12.5% of Chinese adult inpatients suffer from malnutrition (15). Sarcopenia is a key manifestation of malnutrition commonly observed in chronic diseases, including cirrhosis (10, 11). PSVD is a rare but increasingly recognized liver vascular disorder. While sarcopenia is well-documented in cirrhosis, research on PSVD remains limited. In a previous study, 38% of patients with PSVD were classified as having sarcopenia (16), nearly twice the proportion observed in our cohort (17 of 92 patients). This discrepancy may be attributed to differences in sample size (16 vs. 92 PSVD patients) and in the diagnostic criteria used to define sarcopenia. Unlike patients with cirrhosis, more than two-thirds of PSVD patients are asymptomatic and exhibit only subtle clinical or laboratory signs of portal hypertension (6). Therefore, using a cirrhotic control group and applying sarcopenia cut-offs established for cirrhosis is not appropriate. L3-PMI is an established method for evaluating skeletal muscle mass, offering shorter measurement time, potentially lower measurement errors and a strong sensitivity to age, compared to L3-skeletal muscle index (SMI) (14). A positive linear correlation between L3-PMI and L3-SMI was observed in a healthy Chinese population. Therefore, we adopted L3-PMI for sarcopenia diagnosis instead of L3-SMI. In summary, given the relatively well-preserved liver function in our specific Chinese population with PSVD and to reduce the impact of sex differences and the physiological decline of skeletal muscle with age, we adopted age- and sex-specific L3-PMI values using the 5th-percentile threshold for defining sarcopenia (14). Given that poorer performance of PMI was observed in patients with cirrhosis compared to SMI (17), further study is needed to evaluate the comparative predictive performance of L3-PMI and L3-SMI in patients with PSVD. Among symptomatic patients, EGVB is a common portal hypertension-related complication in PSVD. In our cohort, EGVB was the predominant manifestation (65.22%), occurring either alone or in combination with moderate-to-severe ascites. In this context, the significant differences in hemoglobin and white blood cell levels between the Sar + and Sar- groups may be attributable to EGVB. Despite the high prevalence of underlying disorders in the cohort, liver function was comparable between the Sar + and Sar- groups. However, the Sar + group exhibited significantly higher rates of moderate-to-severe ascites and EGVB. A lower L3-PMI was associated with decompensation events but not with Child-Pugh stage, thereby linking sarcopenia more strongly to portal hypertension than to underlying liver function or specific etiologies.

Currently, the optimal management strategy for EGVB related to PSVD remains poorly defined. The recommended strategy for managing EGVB generally aligns with that for cirrhosis (8, 9). In our cohort, 60 patients had a history of EGVB: 23 were managed with TIPS and 37 with endoscopic therapy. Current clinical guidelines on TIPS identify sarcopenia as a potential predictor of post-TIPS outcomes (18). A retrospective study reported that TIPS placement significantly improved skeletal muscle index and mitigated sarcopenia (12). Consistently, an important finding of our study was that L3-PMI significantly increased after TIPS placement. Thus, portal hypertension likely plays a key role in the development of sarcopenia in PSVD patients. However, this analysis has several limitations, including a small sample size (*n* = 23; sarcopenia subgroup *n* = 4), lack of endoscopic treatment controls, and absence of comprehensive nutritional assessments (e.g., handgrip strength, timed chair stand test). Therefore, the effect of TIPS on nutritional status in patients with PSVD needs to be further validated in prospective studies. Notably, few studies have evaluated the efficacy and safety of standard therapies and TIPS for EGVB in PSVD patients (19, 20). While combined endoscopic/NSBBs therapy versus TIPS comparisons are common in cirrhosis studies, such data are lacking for PSVD (21–23). Our study first revealed contrasting but non-significant trends: endoscopic treatment showed a higher 1-year rebleeding rate than TIPS, whereas TIPS was associated with a higher 1-year incidence of OHE. A randomized controlled trial with an adequate sample size and sufficient follow-up duration is warranted to definitively compare endoscopic therapy versus TIPS in PSVD.

Sarcopenia has been shown to adversely affect clinical outcomes in cirrhotic patients (24). In patients with cirrhosis undergoing TIPS, sarcopenia has emerged as a key predictor of post-TIPS hepatic encephalopathy, independent of conventional prognostic factors (25, 26). Consistently, our results showed an increasing trend of OHE among patients with sarcopenia. Although statistical significance was not reached, our findings underscore the importance of routinely assessing sarcopenia and nutritional status before TIPS in patients with PSVD. In cirrhosis patients with EGVB, more than 15% experience rebleeding despite standard treatment, which is associated with a 6-week mortality rate of approximately 40% (18). Sarcopenia has been identified as an independent risk factor for 1- and 2-year rebleeding in cirrhotic patients undergoing endoscopic treatment (10, 11). In the time-dependent analysis, the Sar + group exhibited a higher cumulative incidence of 1-year rebleeding compared to the Sar- group. According to the multivariable Cox regression analysis, sarcopenia was an independent predictor of variceal rebleeding. Based on our findings, we speculate that EGVB and sarcopenia may reinforce each other in a bidirectional manner, thereby creating a vicious cycle. Each episode of EGVB may worsen nutritional status, whereas sarcopenia further increases the risk of rebleeding, hospitalization, and other adverse clinical outcomes, potentially through systemic inflammation and heightened susceptibility to infection. Moreover, sarcopenia may serve as a clinical surrogate for the severity of portal hypertension. On the one hand, because hepatic function is typically well preserved in patients with PSVD, sarcopenia in this population may be driven primarily by portal hypertension and only minimally influenced by intrinsic liver dysfunction. On the other hand, an increased risk of variceal rebleeding occurs in cirrhotic patients with clinically significant portal hypertension, defined as a hepatic venous pressure gradient ≥10 mmHg (8), supporting a potential association between sarcopenia and portal hypertension degree. Notably, a previous study reported that sarcopenia increases mortality in cirrhosis, independent of the severity of portal hypertension (27). Future studies are needed to clarify whether the severity of sarcopenia reflects the degree of portal hypertension in PSVD. Moreover, similar to the management of cirrhosis, nutrition support should be initiated as early as possible for patients with PSVD (8).

This study has several limitations. First, the retrospective design of this study and inclusion of PSVD patients with clinically significant portal hypertension and available CT imaging, may introduce selection bias. Second, sarcopenia was diagnosed using cut-offs values established in a northern Chinese population, whereas our cohort was from southwestern China. Regional differences in environmental factors and lifestyle may represent potential diagnostic bias. Third, the modest sample size, relatively short follow-up duration, and inherently favorable prognosis of PSVD make it difficult to assess long-term prognostic differences. Finally, the severity of sarcopenia was not stratified, and the comparative predictive performance of L3-PMI and L3-SMI for adverse clinical outcomes was not evaluated.

## Conclusion

Sarcopenia is prevalent in patients with PSVD and appears to be independently associated with a 1-year risk of rebleeding in those with a history of EGVB and endoscopic treatment. A comprehensive nutritional assessment should be incorporated into the routine management of PSVD patients. Our preliminary findings suggest that TIPS creation may improve muscle mass in PSVD patients, which is consistent with earlier findings in cirrhosis. Prospective, well-designed studies are warranted to directly compare TIPS with endoscopic therapy for the management of EGVB in the PSVD population.

## Data Availability

The datasets presented in this article are not readily available because the data are not publicly available due to privacy or ethical restrictions. Data available on request from the corresponding author. Requests to access the datasets should be directed to hxxhwh@163.com.
